# Dynamic combinatorial chemistry directed by proteins and nucleic acids: a powerful tool for drug discovery

**DOI:** 10.1039/d5cs00223k

**Published:** 2025-07-08

**Authors:** Antonio Aguanell, Marc Hennebelle, Miguel Ángel Ortega, Ruth Pérez-Fernández

**Affiliations:** a Molecular and Cellular Biosciences Department, Centro de Investigaciones Biológicas Margarita Salas, CIB-CSIC Madrid 28040 Spain ruth.perez@csic.es; b Departamento de Química Física, Universidad Complutense de Madrid, 28040 Madrid Spain

## Abstract

Protein-directed dynamic combinatorial chemistry (P-D DCC) is a powerful strategy for identifying ligands to protein targets of pharmacological significance. It leverages a thermodynamic templated effect, where proteins selectively amplify high-affinity binders. In contrast, although nucleic acids play critical roles in gene regulation and disease and offer significant therapeutic potential, they remain underexplored in drug discovery. While P-D DCC has been widely applied, the use of nucleic acid-directed dynamic combinatorial chemistry (NA-D DCC) is relatively limited. Expanding these methodologies is essential for tackling emerging infectious diseases and advancing therapeutic development. This review examines the applications, experimental design considerations, recent advancements, and P-D DCC and NA-D DCC perspectives.

## Abstract 2

Dynamic combinatorial chemistry meets proteins and nucleic acids: a path for drug discovery.

## Graphical abstract

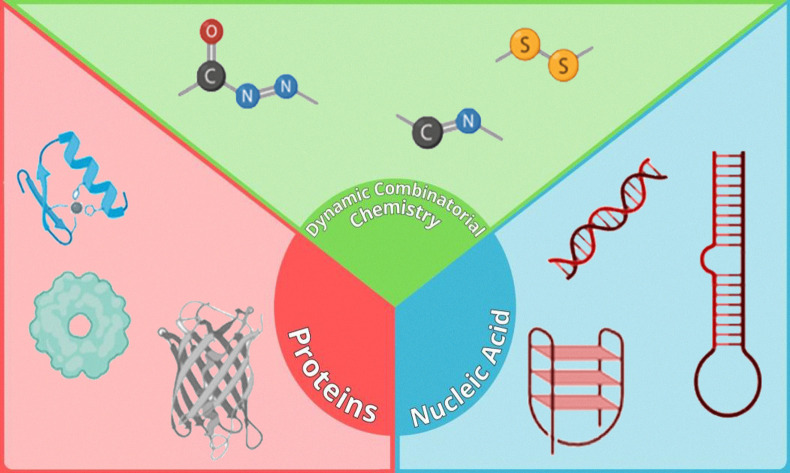

## 1 Introduction

Over the past decade, significant progress has been made in developing efficient lead compounds for biological targets through strategies such as high-throughput screening, rational design, and, increasingly, fragment-based approaches. Among emerging methodologies, template-directed chemical synthesis has proven to be a powerful tool for accelerating drug discovery. Using pharmacologically relevant targets as templates facilitates the one-step synthesis and screening of multiple molecular libraries, yielding high-affinity ligands.

**Fig. 1:**
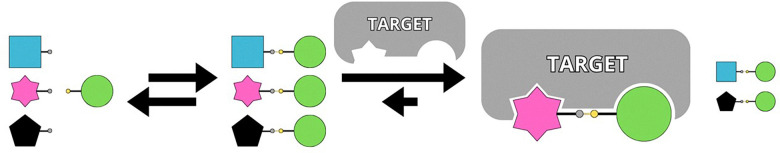
General scheme of a protein or nucleic acid-directed DCC.

Within the various template-driven synthetic strategies, molecular recognition in template-directed dynamic combinatorial chemistry (DCC) occurs within a thermodynamically controlled dynamic combinatorial library (DCL). This system enables the self-correction of bonds between library members in response to a biological template. When one or more molecules in the mixture bind to form a stable complex with the template, equilibrium shifts according to Le Chatelier's principle, amplifying the preferred compound at the expense of others in the library (Fig. 1).^1–3^

Unlike kinetic target-guided synthesis (KTGS),^3,4^ such as *in situ* click chemistry, where the template accelerates an irreversible reaction by stabilizing a ternary complex between two reactive building blocks, target-directed dynamic combinatorial chemistry (T-D DCC) relies on thermodynamic control. In T-D DCC, weaker binders are naturally eliminated, while stronger binders are favored and maintained, regardless of how quickly they initially formed. A key advantage of T-D DCC is its ability to operate with substoichiometric amounts of the target, allowing actual competition among potential binders. Both KTGS and T-D DCC support the exploration of alternative or unexpected protein conformations by preserving the protein's conformational flexibility.

This review highlights the advances in protein- and nucleic acid-directed DCC over the past fifteen years, emphasizing experimental setups and applications. Ref. 1,2 provide foundational insights into protein-directed DCC, while ref. 3 focuses on aspects related to nucleic acid-directed DCC.

## 2 Designing a successful templated dynamic combinatorial system

A successful templated DCC experiment requires four key components: a protein or nucleic acid template, a biocompatible reversible chemistry, a diverse set of building blocks (BBs) as library components, and a suitable analytical technique tailored for the library of interest.

### 2.1 Protein and nucleic acid templates

Ensuring the native state of the target is essential for obtaining reliable experimental results in dynamic combinatorial chemistry (DCC). Ideally, dynamic combinatorial libraries (DCLs) should be assembled in an aqueous buffer with a minimal concentration of an organic co-solvent (*e.g.*, DMSO, MeOH) to prevent building block precipitation. The proportion of organic solvent must be optimized based on the tolerance of the biological template, as excessive concentrations may induce structural perturbations or precipitation.

Template precipitation results in its removal from the DCL, while even subtle alterations in its three-dimensional structure can lead to the selection of ligands against a modified target. Given that pH, temperature, buffer composition, ionic strength, and specific ions influence template stability, a thorough assessment of the template's structural integrity under DCL conditions is strongly recommended before experimentation.

The template concentration must align with the DCL building blocks to ensure effective competition among dynamic combinatorial library (DCL) members for the template. Additionally, the stability of the template under DCL conditions dictates whether an adaptive or pre-equilibrated DCL approach is employed. In an adaptive DCL, equilibration occurs in the presence of the template, allowing for the continuous selection of the most favorable binding partners. Conversely, when dealing with low-stability templates, the DCL is pre-equilibrated in the absence of the template and subsequently re-equilibrated upon its addition.^5,6^ Notably, the majority of examples reviewed here correspond to adaptive DCLs.

It is essential to highlight that the examples presented in Table 1 are from 2019 onward, while earlier cases can be found in previous reviews.^1–3,7^

**Table 1:** Some reported examples of protein and nucleic acid-directed DCC

Reversible exchange	Protein target	Analytical method	DCL BBs	DCL conditions	Ref.
Ester	BSA	SEC-MS	(4 × 2), (1 × 4)	Microfluidic system	(He *et al.*, 2019)^8^
Ester	BSA	SEC-MS	24 × 22	Microfluidic system	(Qiu *et al.*, 2019)^9^
Oxime	mGAT1	LC-MS	22 × (4 × 1)	PBS buffer, pH 7.1	(Kern *et al.*, 2019)^10^
Acylhydrazone	14-3-3 protein	LC-MS	3 × 6	MES buffer, pH 6.5, cat. aniline, r.t.	(Hartman *et al.*, 2020)^11^
Acylhydrazone	α-Glucosidase and α-amylase	LC-MS	(5 × 3) × 2	PBS buffer, pH 6.25, cat. aniline, r.t.	(Wu *et al.*, 2022)^12^
Acylhydrazone	Cease	LC-MS	(6 × 3)	Ammonium acetate buffer, pH 6.2, cat. aniline, r.t.	(Zhao *et al.*, 2021)^13^
Acylhydrazone	NCS-1/Ric8a complex	STD-NMR	(1 × 5)	Tris buffer, pH 7.4, cat. *p*-anisidine, 4 °C	(Canal-Martín *et al.*, 2019)^14^
Acylhydrazone	RAGE	MST	(4 × 6), (5 × 4)	PBS buffer, pH 6.9, cat. aniline, r.t.	(Dascalu *et al.*, 2024)^15^
Acylhydrazone	IspE	LC-MS	6 × 12	Tris buffer, cat. aniline	(Braun-Cornejo *et al.*, 2024)^16^
Acylhydrazone	DXS	LC-MS	(3 × 7) × 2, (15 × 2), (11 × 2), (3 × 8)	PBS buffer, pH 6.25, cat. aniline, r.t.	(Jumde *et al.*, 2021)^17^
Acylhydrazone	ECF-PanT	LC-MS	2 × 7	PBS buffer, pH 7.5, cat. aniline	(Exapicheidou *et al.*, 2024)^18^
Acylhydrazone	Urease	LC-MS	(5 × 3) × 2	PBS buffer, pH 6.25, cat. aniline, r.t.	(Wu *et al.*, 2022)^19^
Acylhydrazone	RAD51-BRCA2	LC-MS	13 × 9	HEPES buffer, pH 7.02, cat. aniline	(Bagnolini *et al.*, 2022)^20^
Acylhydrazone	Nsp10, Nsp10–Nsp14 and Nsp10–Nsp16	LC-MS	(3 × 7), (3 × 8)	PBS buffer, pH 7.04, cat. aniline, r.t.	(Jumde *et al.*, 2025)^21^
Acylhydrazone	PHD2	FP	5 × 90	HEPES buffer, cat. *p*-anisidine	(Li *et al.*, 2022)^22^
Acylhydrazone	AChE	LC-MS	1 × 5	PBS buffer, pH 6.2, cat. aniline	(Xu *et al.*, 2020)^23^
Acylhydrazone	Pentameric *E. coli* heat labile enterotoxin B subunit (LTB)	LC-MS	1 × 5	PBS buffer, pH 6.2, cat. aniline, r.t.	(Xu *et al.*, 2020)^23^
Acylhydrazone	BChE	LC-MS	1 × 6	PBS buffer, pH 6.2, cat. aniline, r.t.	(Zhao *et al.*, 2021)^13^
Acylhydrazone	G-quadruplex (G4) DNA	HPLC-MS, CD, fluorescence	2 × 3	Ammonium acetate buffer, pH 6.4	(Reznichenko *et al.*, 2021)^24^
Imine	CA	^1^H-NMR	1 × 1 × 1	D_3_PO_4_–NaOD buffer, pD 9.0, 40 °C for 2 days	(Zhang *et al.*, 2020)^25^
Imine	HIV-TAR and RRE-IIB RNA	ESI-MS	6 × 1	Tris buffer, pH 6.3	(Umuhire *et al.*, 2020)^26^
Imine	G-quadruplex (G4) DNA and duplex DNA (dsDNA).	HPLC-MS, CD, fluorescence	10 × 1	MOPS buffer, pH 6.5	(Jana *et al.*, 2019)^27^

### 2.2 Dynamic combinatorial library building blocks

The selection of building blocks is critical to the success and outcome of a dynamic combinatorial library (DCL) design. Building blocks must possess functional groups capable of undergoing reversible exchange according to the chosen chemistry, exhibit complete solubility under DCL conditions, and display structural and geometric diversity.

When prior knowledge of ligands for the target or structural information is available, a “warhead” strategy can serve as a rational starting point for DCL design. In this approach, a building block known to interact with the target is functionalized with a reversible-reacting group, allowing it to conjugate with other library members. This strategy facilitates the exploration of additional binding sites, such as adjacent protein pockets or oligonucleotide loops, enhancing the likelihood of identifying high-affinity ligands.

It is also advisable to select isoenergetic building blocks to minimize energetically unfavorable equilibrium shifts. This reduces biases in the reaction mixture that may otherwise predispose the system to form specific products preferentially.

In cases where structural information is unavailable to inform building block selection, various chemical navigators and privileged structure databases can serve as valuable resources for guiding library design.^28^ These structures represent privileged scaffolds, frequently emerging as key hits in medicinal chemistry.

To address the increasing challenges of analyzing complex systems, the Miller group developed resin-bound dynamic combinatorial chemistry (RB-DCC)—a technique designed to overcome the analytical limitations of solution-based systems while preserving the broad testing capabilities of dynamic combinatorial libraries (DCLs). RB-DCC employs spatially segregated, resin-immobilized building blocks that dynamically interact with solution-phase components in the presence of a target. This approach facilitates the rapid identification of high-affinity ligands by leveraging fluorescence microscopy and mass spectrometry, significantly enhancing library deconvolution and ligand discovery efficiency.^29,30^

However, RB-DCC has inherent limitations, including (a) the requirement for fluorescently labelled biomolecules, which may introduce steric or electronic perturbations affecting binding interactions, (b) limitation in the choice of building blocks to those that are either non-fluorescent or have fluorescence emission that does not overlap with that of the labeled target.

### 2.3 Exchange reactions and “catalysts” for accelerating dynamic covalent equilibration

The adaptability of a dynamic combinatorial library (DCL) fundamentally depends on the choice of reversible chemistry. The selected chemical reactions must meet the following criteria: (i) efficiency and reversibility within a reasonable time frame under DCL conditions (*e.g.*, appropriate pH and temperature), (ii) mild reaction conditions to prevent disruption of the molecular recognition process, ensuring compatibility with biological templates, (iii) controlled termination to facilitate the analysis and isolation of amplified compounds. This can be achieved through strategies such as imine reduction to stable amines using NaBH_3_CN or acidification to halt disulfide exchange equilibration.

Table 2 summarizes the latest biocompatible and extensively studied reversible chemistries, highlighting the optimal conditions for efficient exchange.

**Table 2:** Reversible chemistries applied in protein- and nucleic acid-directed DCC and their reaction conditions

Reversible exchange name	General conditions
Metal	pH ∼ 7. Mild aqueous conditions.
Ester	Catalyzed by acids or bases.
Nitroaldol	Basic conditions (pH > 7). Often catalyzed by amines or metal-based catalysts.
Thio-Michael	pH ∼ 7. Mild aqueous conditions.
Thioester	Operates at pH ∼ 7. Mild aqueous conditions
Boronic acid/boronate ester exchange	pH > p*K*_a_ of boronic acids.
Hemithioacetal	pH > 7. Mild basic conditions.
Imine	pH range (4–7). Post-reduction with NaBH_3_CN.
Oxime	pH range (4–8). A catalyst is needed under moderately basic conditions.
Hydrazone	pH range (4–8). A catalyst is needed in moderately basic conditions.
Acylhydrazone	pH range (4–8). A catalyst is needed in moderately basic conditions.
Acylhydrazone	pH range (4–8). A catalyst is needed in moderately basic conditions.
Disulfide	pH ≥ 7. Catalyst needed for faster equilibration under certain conditions.

#### 2.3.1 Noncovalent bond reactions

##### 2.3.1.1 Metal ligand exchange

**Fig. 2:**
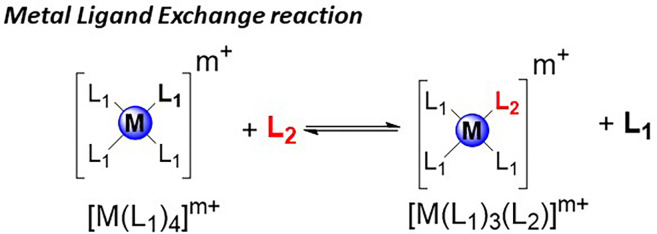
General reaction showing the metal ligand exchange used in DCC.

The reaction rates are influenced by the metal type, its oxidation state, and the steric and electronic properties of the coordinating ligands.^31^ Consequently, the experimental conditions necessary for efficient exchange vary significantly depending on the metal and ligand type. Various well-established methods are available to freeze the library and halt the exchange process, including modulation of the metal center's oxidation state through oxidation,^32^ or the library members can be covalently captured^33^ (Fig. 2).

One drawback of this exchange lies in the lability of the metal–ligand interactions, which can hamper the isolation of library members. However, this is no longer an issue if the number of noncovalent connections within the complex is large. This exchange has been effectively applied to protein and nucleic acid DCLs.^34,35^

#### 2.3.2 Covalent bond reactions

##### 2.3.2.1 Acyl exchange

**Fig. 3:**
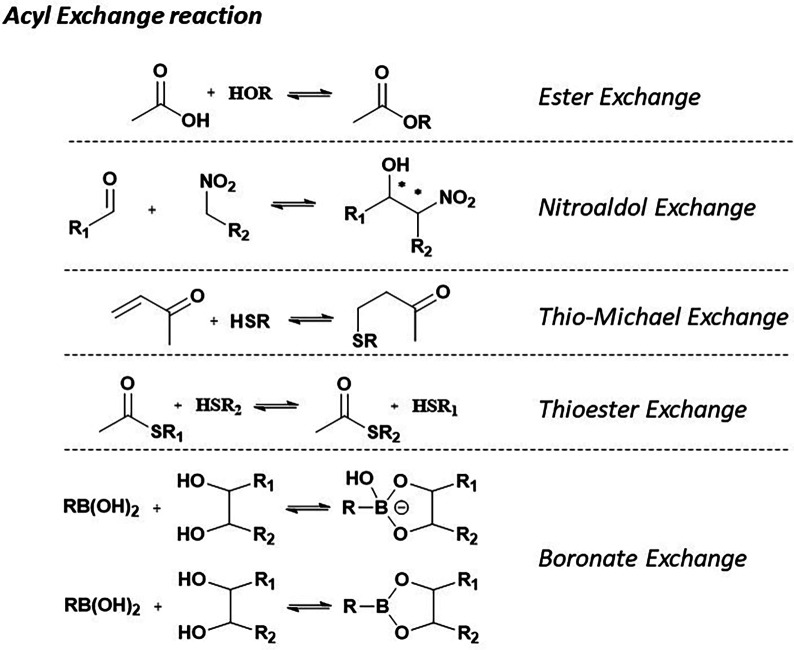
General reactions showing the use of acyl exchange in DCC.

Acyl exchange occurs in dynamic combinatorial libraries under mild conditions, depending on the reaction's components, and in neutral conditions at ambient temperature. C(O)–X bonds are relatively fragile and reversible when X is an electronegative fragment (*e.g.*, O, S, or N) (Fig. 3).

###### 2.3.2.1.1 Ester exchange

Ester-based substrates are commonly used, forming dynamic yet moderately stable bonds responsive to equilibrium shifts. An example of this was reported by Sanders and colleagues years ago, who prepared derivatives of quinine and cinchonidine, each featuring a hydroxy group at one end and a methyl ester group at the other.^36,37^

###### 2.3.2.1.2 Aldol/nitroaldol exchange

Aldol formation in dynamic combinatorial chemistry (DCC) occurs under mildly to moderately basic conditions (pH 8.0–10.5), typically at room temperature, to minimize undesired side reactions. In this reversible process, aldehydes or ketones serve as electrophiles, undergoing condensation with enolate anions to produce β-hydroxy carbonyl compounds. This increases the complexity and branching of dynamic combinatorial libraries (DCLs).

Despite its potential, aldol formation has been relatively underexplored in protein-directed dynamic combinatorial chemistry (P-D DCC).

A catalytic system featuring 1,5,7-triazabicyclo[4.4.0]dec-5-ene (TBD) and neutral alumina showed efficient exchange dynamics, with the equilibrium effectively halted upon catalyst removal. Furthermore, the system displays solvent-dependent selectivity and stereoisomeric preference, underscoring its potential for tuning molecular diversity.^38^

Vongvilai *et al.* reported an example of using an enzyme to catalyze a nitroaldol reaction, generating a dynamic combinatorial library (DCL).^39^

###### 2.3.2.1.3 Thio-Michael exchange

The thio-Michael exchange is mechanistically related to acyl transfer reactions and involves the reversible addition of thiols to Michael acceptors. This reaction integrates several advantageous features, including rapid equilibration, reversibility under physiological conditions, and mild reaction parameters, making it highly compatible with aqueous environments and pH-responsive systems.

The biological relevance of thiol addition reactions is exemplified by glutathione-mediated detoxification, catalyzed by glutathione-*S*-transferase enzymes. This underlines the versatility of this approach for designing dynamic systems. At pH eight and room temperature, the thio-Michael reaction reaches equilibrium within 30 minutes, ensuring efficient DCL assembly and screening.

Additionally, the reaction offers precise dynamic control. It remains reversible under basic conditions while “switching off” at acidic pH, allowing tailored regulation of library dynamics. Its compatibility with physiological environments, proceeding under mild aqueous conditions, facilitates direct interfacing with proteins and biomacromolecules, making it a highly promising tool for biological applications in dynamic combinatorial chemistry.^40^

###### 2.3.2.1.4 Thioester exchange

The thiol-thioester exchange reaction is typically carried out by mixing stoichiometric amounts of thiols and thioesters in an aqueous solution. This exchange occurs under neutral conditions at room temperature and has been widely applied in peptide chemistry to facilitate coupling reactions.^41^ A notable example is the work by Woll and Gellman, who replaced an amide bond with a thioester in bovine pancreatic polypeptide (bPP) to enable rapid and reversible (dynamic) exchange of the α-helical segment with other thiols in solution. To prevent disulfide formation, the authors employed tris-carboxyethylphosphine (TCEP) as a reducing agent, ensuring controlled exchange dynamics.^42^

###### 2.3.2.1.5 Boronic acid/boronate ester exchange

Reversible boronic acid-mediated reactions have emerged as a fundamental component of dynamic combinatorial chemistry (DCC), with extensive applications in chemical biology and biomedical science. A key feature of this approach is the reversible formation of boronate esters, primarily through the interaction of boronic acids with diols. These esters exhibit exceptional adaptability under physiological conditions.

Over the past decade, the scope of boronic acid-based DCC has expanded to encompass more sophisticated chemistries, including iminoboronate and salicylhydroxamic–boronate conjugates, broadening its applicability in ligand discovery and molecular recognition. Furthermore, boronate ester formation's kinetic and thermodynamic tunability allows precise equilibrium control, ensuring compatibility with aqueous systems across a range of pH levels. This versatility enhances its potential for biological applications and synthetic methodologies, making boronic acid-mediated reactions a valuable tool in DCC.^43^

##### 2.3.2.2 Acetal exchange

###### 2.3.2.2.1 Hemiacetal exchange

**Fig. 4:**
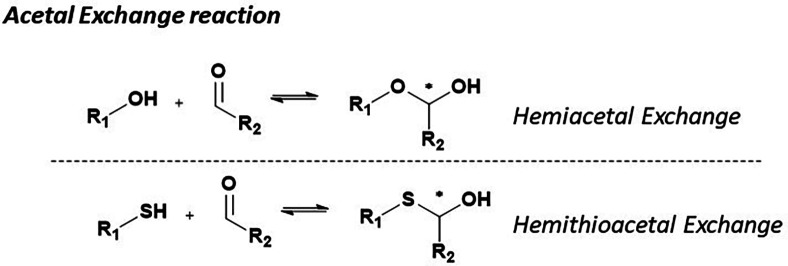
General schemes of acetal exchange reactions in DCC.

Hemiacetal formation and exchange occur under mildly acidic to neutral conditions (pH 5.5–7.5) at room temperature, where ketones react with alcohol to generate dynamic, reversible hemiketal linkages. Ketones are particularly advantageous in dynamic combinatorial libraries (DCLs) due to their capacity to form relatively stable yet reversible hemiketal bonds, creating structurally diverse systems (Fig. 4).

Hemiacetals are formed through the reversible condensation of alcohols with carbonyl groups, though they typically exhibit limited stability unless structurally stabilized, as observed in cyclic carbohydrates. Despite their transient nature, hemiacetals offer rapid exchange kinetics, allowing a quick equilibrium within a DCL. Their formation can be enhanced by protonation or metal cation coordination, enabling precise control over equilibrium dynamics and selectivity within the library. This tunability is particularly valuable for applications requiring controlled release mechanisms or selective molecular recognition.^44^

###### 2.3.2.2.2 Hemithioacetal exchange

HTAs are formed through the rapid and reversible condensation of thiols with aldehydes or ketones to create hemithioacetals, establishing a dynamic system that continuously regenerates its components under neutral to basic conditions (pH 7–9). Like hemiacetals, ketones are often favored for use in these systems due to their ability to form relatively stable yet reversible hemithioacetal linkages, suitable for generating diverse structures in DCLs^37,45^ (Fig. 4).

##### 2.3.2.3 C=N exchange

**Fig. 5:**
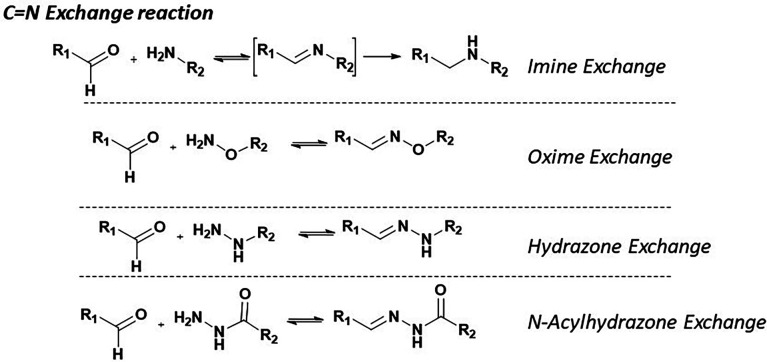
General reactions showing the use of Ca-on nitrogen double bond formation used in DCC.

The choice of nitrogen source significantly influences the kinetic and thermodynamic stability of the resulting carbonyl derivatives. Imine formation involves the condensation of primary amines with aldehydes and is kinetically favored, but it is often thermodynamically unstable, particularly in aqueous environments, and is prone to hydrolysis. This inherent transience limits its utility in stable dynamic combinatorial libraries (DCLs) unless further stabilized, such as through reductive amination to form secondary amines (Fig. 5).

Alternative nitrogen-containing compounds, including hydroxylamines, hydrazines, and acylhydrazides, are frequently utilized to address these stability challenges. These reagents facilitate the formation of oximes, hydrazones, and acylhydrazones. The resulting derivatives exhibit enhanced hydrolytic resistance, making them more suitable for dynamic exchange processes while maintaining C=N bond reversibility. Oximes demonstrate high hydrolytic stability, attributed to intramolecular hydrogen bonding from the –OH group. This shields the imine bond from nucleophilic attack. Hydrazones benefit from the stabilizing influence of their –NH group, and *N*-acyl hydrazones exhibit further enhanced stability due to an electron-withdrawing acyl group, strengthening the C=N bond.

These stabilized imine analogs provide a versatile and tunable platform for building DCLs, enabling molecular recognition in biologically relevant environments.^46^

###### 2.3.2.3.1 Imine exchange

Imine formation occurs primarily at mildly acidic to neutral pH (typically 5.0–7.0) and room temperature, favoring the reversible condensation of primary amines with aldehydes or ketones to form C=N bonds. Aromatic aldehydes are preferred as they lead to more stable imine adducts. To capture and analyze specific equilibrium states in the dynamic library, a reducing agent such as NaBH_3_CN is often added at the end of the reaction to freeze the equilibrium and convert imines to stable amines for subsequent analysis in case the imines formed are not sufficiently stable.^47–49^

###### 2.3.2.3.2 Oxime exchange

Oxime exchange involves the reaction of hydroxylamines with aldehydes or ketones under slightly acidic to neutral conditions (pH 4.5–7.0) at ambient temperature. Aromatic aldehydes are commonly favored due to their ability to form stable oxime linkages, which are particularly valuable for constructing dynamic combinatorial libraries. Oximes' enhanced kinetic and thermodynamic stability is attributed to electronic contributions from the delocalization of the oxygen pair within the C=N bond. Although oxime formation is slower than imine formation, this reduced reaction rate contributes to stable yet reversible adducts, enabling fine-tuned equilibrium and robust screening processes. Recent studies have highlighted the potential of oxime exchange in covalent adaptable networks.^50^

###### 2.3.2.3.3 Hydrazone exchange

Whereas imines are formed and hydrolyze quickly at neutral to acidic pH but are unstable in water, hydrazones can be thermodynamically stable even at low pH and tend to be kinetically inert under neutral conditions. Hydrazone exchange is primarily achieved under mildly acidic to neutral conditions (pH 5.5–7.5) and at room temperature. It involves the reaction of hydrazines with aldehydes or ketones. Aromatic aldehydes are particularly favored due to the enhanced stability of the hydrazone bonds they form. Compared to imine formation, hydrazone formation occurs more rapidly and yields more stable adducts, making these linkages especially suitable for DCLs that rely on stability and reversible assembly.^51–53^

###### 2.3.2.3.4 *N*-Acylhydrazone exchange

The acylhydrazone equilibrium has emerged as the most widely employed among carbonyl-based reactions and within the P-D DCC reactions. The reversible formation of *N*-acylhydrazones (NAH) through *N*-acylhydrazides reaction with aldehydes or ketones provides a flexible and efficient means of generating DCLs. This equilibrium offers several advantages: reversible under mildly acidic to neutral pH conditions (5.5–7.0) and is compatible with various buffer systems (MES, ammonium acetate, Tris, PBS, HEPES).^11,14,15,54,55^

##### 2.3.2.4 Disulfide exchange

**Fig. 6:**
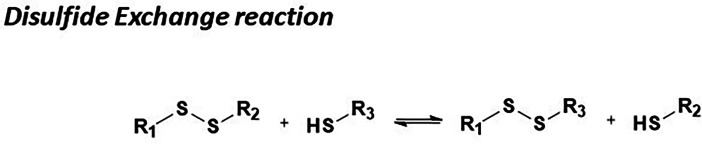
General reaction showing the use of disulfide exchange in DCC.

Disulfide exchange represents a fundamental example of sulfur-based dynamic chemistry, demonstrating exceptional versatility in dynamic combinatorial chemistry (DCC). The mechanism proceeds *via* nucleophilic displacement, wherein a thiolate anion attacks a disulfide bond, displacing another thiolate anion. This process occurs readily under mild to basic aqueous conditions and is highly pH-dependent (Fig. 6).

Dynamic combinatorial libraries (DCLs) can be initiated by free thiols or by activating a disulfide-based DCL by adding a catalytic amount of a reducing agent, such as dithiothreitol (DTT). The exchange persists as long as thiolate anions are present in the solution and can be halted by lowering the pH, leading to the thiolate species' protonation and deactivation.^56^

At low concentrations, stereochemical self-selection promotes meso diastereomer formation. In contrast, at higher concentrations, supramolecular polymerization leads to a reduction in stereoselectivity. Furthermore, disulfide exchange integrates seamlessly with metal coordination and imine chemistry, enabling tunable equilibrium shifts. This adaptability extends its applicability beyond traditional DCC, making it a valuable tool in molecular recognition, systems chemistry, and the design of responsive materials.^57,58^

#### 2.3.3 Exchange catalysts

Despite their potential, the applicability of some of these chemistries is constrained by their relatively slow exchange rates under physiological conditions. For example, (acyl)hydrazone formation predominantly occurs under mildly acidic to neutral conditions (pH 5.5–7.5) at room temperature, which can limit its dynamic responsiveness in specific biological contexts.

**Fig. 7:**
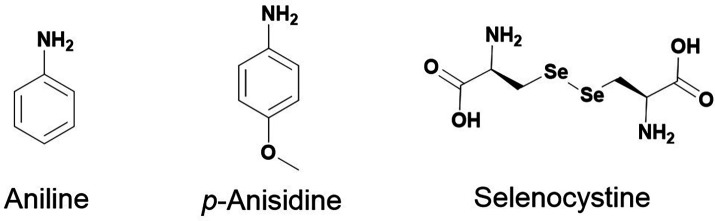
Examples of reported small organic catalysts for DCC reactions.

For the last fifteen years, nucleophilic catalysts have been used to speed up acylhydrazone exchange, including aniline as a catalyst.^11,15–17,54,59,60^ More recently, the biocompatible aniline derivative, *p*-anisidine, has been reported to be more efficient than the aniline as a catalyst (Fig. 7).^14,22,61^

Besides, many other catalysts are used to form *N*-acylhydrazones but have not yet been applied in P-D DCC. For instance, anthranilic acids have been reported as superior catalysts for hydrazone and oxime formation compared to aniline. Moreover, alternative proton donors were examined with varied p*K*_a_, showing that *ortho* proton donor groups strongly enhance the activity of nucleophilic catalysts in hydrazone formation. Besides, tuning the p*K*_a_ of the proton donor toward the biological buffer pH further enhances catalysis.^62,63^ Indolines with withdrawing groups have been reported as a new catalyst scaffold, which can catalyse acylhydrazone, hydrazone, and oxime formation *via* an iminium ion intermediate^64^ as well as hydrazone and oxime peptide ligation in the presence of arginine.^65^

Incorporating selenium compounds, such as selenocystine (Sec_ox_), has significantly enhanced thiol-disulfide dynamic combinatorial chemistry (DCC), overcoming a key limitation—slow equilibration under physiological conditions. Inspired by the catalytic mechanism of the thioredoxin system in mammalian cells, Sec_ox_ functions as an enzymatic mimic, accelerating thiol-disulfide exchange. This enhancement arises from selenium's superior nucleophilicity and electrophilicity, facilitating the exchange process and leaving group ability. These properties stem from selenium's lower bond dissociation energy and p*K*_a_ compared to sulfur, enabling more efficient dynamic exchange. In addition, they enhance reaction rates and broaden the pH range for oxidative folding and exchange reactions. Sec_ox_ has demonstrated remarkable efficiency in P-D DCC, facilitating rapid equilibration within 24 hours—significantly faster than sulfur-based systems, which often require several days. Its versatility is further exemplified by its ability to promote the correct folding of scrambled RNase A at acidic pH.^66^ Furthermore, it doesn’t interfere with the template, as shown in the protein-directed DCC using glucose oxidase as a protein template where a non-competitive inhibitor was amplified.

## 3 Analytical techniques

### 3.1 Analytical techniques for library identification

#### 3.1.1 HPLC-MS (high pressure liquid chromatography-mass spectrometry)

HPLC-MS is the most frequently used technique, applied across multiple types of reversible exchanges. HPLC-MS is valuable for its ability to separate complex mixtures and identify compounds based on their mass. This technique needs a blank DCL without the target to run parallel to the templated one. However, its limitation is the need for a chromatogram with separate signals to get the right species concentration, which could be considered time-consuming and, therefore, a disadvantage of this method. Slight differences between the blank and templated chromatograms are detected using each compound's relative peak area (RPA), the fraction of each peak relative to the total peak area of the blank and templated DCL. The amplification factor can be obtained by dividing RPAtemplated/RPAblank.^11,12,16–20,54,67^

#### 3.1.2 STD-NMR (saturation transfer difference—nuclear magnetic resonance)

This technique is widely used in molecular recognition studies, particularly for studying ligand–receptor interactions in solution.^68^ It exploits the principles of saturation transfer to selectively detect binding interactions between small molecules (ligands) and larger biomolecules (*e.g.*, proteins, nucleic acids, or membranes). STD-NMR provides detailed insights into ligand binding affinity, binding epitope mapping (identifying ligand regions involved in interactions), and screening potential drug candidates in fragment-based drug discovery. The method is highly sensitive, requires minimal sample preparation, and is compatible with complex biological matrices, including cell lysates and membrane proteins. Additionally, because it is based on transient interactions, it is well-suited for studying weak and moderate binding affinities, often relevant in dynamic biological systems. It has been reported in DCC to detect the library members in close contact with the protein.^14,45,69^

#### 3.1.3 SEC-MS (size exclusion chromatography-mass spectrometry)

This technique separates compounds based on their size and provides mass data, which helps identify large biomolecules and their complexes with ligands without prior treatment.^8,9^

### 3.2 Analytical techniques for binding affinity quantification

#### 3.2.1 MST (microscale thermophoresis)

It allows for the study of molecular interactions in solution without immobilization, which can assist in measuring binding affinities within DCLs.

It is widely used for studying molecular interactions in solution. MST detects binding affinities by measuring changes in the spatial distribution of fluorescent signals induced by a temperature gradient generated with an infrared laser. The MST signal combines thermophoresis and temperature-related intensity changes (TRIC), which respond to physicochemical changes in the molecular environment, such as ligand binding. MST offers several advantages, including high sensitivity, low sample requirements, and the ability to analyze interactions across a wide affinity range, from picomolar to millimolar. However, MST has limitations. It requires fluorescence labeling, which might alter the protein function, and temperature sensitivity can also impact measurement accuracy. Despite these drawbacks, MST complements techniques like fluorescence polarization and Förster resonance energy transfer, making it a valuable tool for orthogonal validation and high-throughput screening in drug discovery.^15,70^

#### 3.2.2 Differential scanning fluorimetry (DSF)

In the context of dynamic combinatorial chemistry (DCC), differential scanning fluorimetry (DSF) is a valuable tool for screening ligand binding, assessing molecular stabilization, and guiding the selection of dynamic combinatorial libraries (DCLs) toward biologically relevant targets. By monitoring thermal shifts across different DCL conditions, DSF helps refine library composition by favoring species that exhibit stronger stabilization effects on the target biomolecule.

It's a non-destructive and label-free method and requires minimal sample preparation. It applies to weak and transient interactions.

DSF is especially useful in early-stage drug discovery, functioning as a high-throughput method for screening compound libraries and optimizing protein conditions for stability, refolding, and crystallization.^55,71^

#### 3.2.3 FP (fluorescence polarization)

This technique measures the change in fluorescence polarization upon ligand binding, making it useful for high-throughput screening and quantifying interactions in DCLs. A fluorophore-labelled ligand is excited with plane-polarized light, and the emitted light retains polarization if the molecule is large and rotates slowly. In contrast, rapid rotation of a minor, free ligand leads to depolarized emission. The fluorescent ligand binding to a larger target (*e.g.*, protein, biomolecule, or macromolecular assembly) increases polarization, providing fluorescence with a quantitative readout of binding interactions. It applies to various targets, such as proteins and nucleic acids.^22,72^

## 4 Protein-directed dynamic combinatorial chemistry (P-D DCC)

This section has been divided into two sections based on the molecular size of the building blocks composing DCLs: small molecules and polymer-based DCLs. We have considered small molecules with molecular weights ranging from 250 to 1000 Da.

### 4.1 Small molecules DCLs

Dynamic combinatorial chemistry (DCC) is primarily a hit identification approach employed in the early stages of the drug discovery and development pipeline. The structural motifs or pharmacophores identified from DCC libraries serve as valuable starting points for developing more refined drug candidates. While initial hits may have relatively high molecular weights, subsequent lead optimization can focus on reducing molecular size and improving drug-like properties through structure–activity relationship (SAR) studies. Furthermore, using smaller building blocks and applying iterative library design can guide the selection toward compounds with more favorable physicochemical profiles.

Ultimately, DCC is valuable for identifying novel binding motifs, which can then be developed into viable therapeutic leads.

#### 4.1.1 Ester exchange

In recent years, advancements have broadened the application of dynamic combinatorial libraries (DCLs) by integrating esterification reactions within microfluidic systems. This approach combines dynamic combinatorial chemistry (DCC) with cutting-edge microfluidic technology to enhance library complexity and specificity. For example, the microfluidic synthesis of fatty acid esters has demonstrated how DCC can dynamically modulate chemical equilibria to favor the formation of bioactive compounds with high binding affinities to target proteins. In this system, esterification reactions were conducted in microreactors with small diameters, significantly improving mass transfer and mixing efficiency. The system reaches equilibrium in the presence of the target protein, enabling the identification of high-affinity binders.^8,9^

**Fig. 8:**
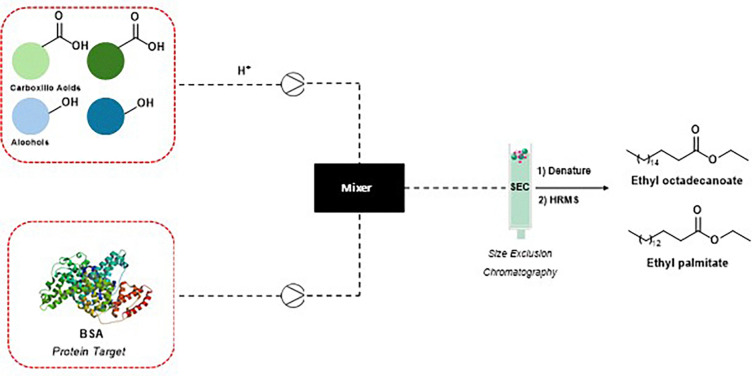
Microfluidic system SEC-HRMS to identify BSA inhibitors.

A novel protocol that integrates microfluidic synthesis with SEC-HRMS analysis was developed, allowing for the identification of protein binders from extensive DCLs (Fig. 8). This approach successfully identified BSA inhibitors, specifically ethyl palmitate and ethyl octadecanoate. Ethyl octadecanoate showed strong binding to BSA, with a binding constant of 4.95 × 10^4^ L mol^−1^ and one primary binding site (*n* ≈ 0.95). Fluorescence quenching studies confirmed a static quenching mechanism, consistent with complex formation. Ethyl palmitate exhibited a similar profile (*K* = 5.21 × 10^4^ L mol^−1^, *n* ≈ 1.03), indicating that both compounds interact effectively with the target protein under physiological conditions.^9^ BSA inhibitors’ therapeutic potential lies in their ability to modulate albumin-related interactions in drug transport and disease pathology. BSA is frequently used as a model for human serum albumin, a crucial carrier of hormones, fatty acids, and drugs in the bloodstream.

#### 4.1.2 Aldol/nitroaldol exchange

**Fig. 9:**
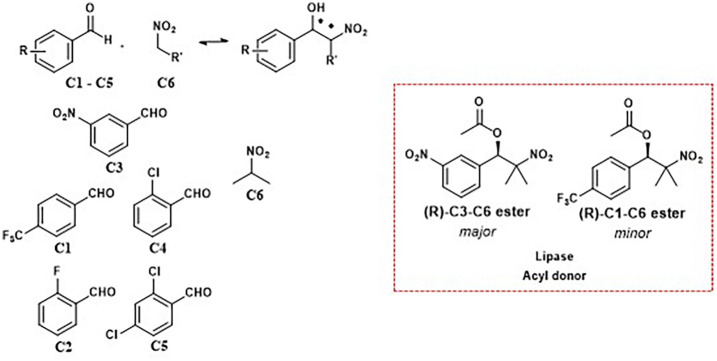
Lipase PSC 1 directed dynamic combinatorial library enabling asymmetric amplification and an enantiomerically enriched product selection.

A notable example of protein-directed aldol exchange is the study by Vongvilai *et al.*, which utilized a lipase to catalyze a nitroaldol reaction, generating a dynamic combinatorial library (DCL) based on carbon–carbon bond formation.^39^ In this work, lipase-catalyzed acylation led to asymmetric amplification, where a specific nitroaldol adduct was preferentially selected as the substrate for enzyme-mediated transesterification. This asymmetric resolution process proved highly effective, yielding the C3–C6 and C1–C6 esters with excellent enantiomeric excess (99% ee and 98% ee, respectively). These results show that combining dynamic nitroaldol libraries with lipase-mediated kinetic resolution allows for the selective amplification and isolation of enantiomerically pure products. The lipase PS-C I from *Pseudomonas cepacia* showed high stereoselectivity, confirming that this approach enables the synthesis of chiral compounds suitable for biological applications (Fig. 9).^39^

#### 4.1.3 Thio-Michael exchange

The conjugate addition of thiols to enones represents a promising strategy for identifying inhibitors of glutathione *S*-transferases (GSTs), a family of enzymes crucial for detoxification and implicated in drug resistance, particularly in cancer and parasitic diseases. By employing glutathione (GSH) and the enone ethacrynic acid, researchers specifically targeted both the GSH binding site and the hydrophobic region of the GST active site. The presence of GST facilitated the amplification of the most potent binding components, ultimately leading to the identification of two novel inhibitors of *Schistosoma japonicum* GST. This enzyme-directed approach integrates chemical synthesis with biological binding assays, enabling the *in situ* selection of highly effective inhibitors. The identified compounds showed low micromolar inhibitory activity and were preferentially amplified despite having similar potency to other library members, demonstrating the system's sensitivity to subtle differences in binding affinity, supporting the use of dynamic combinatorial chemistry (DCC) as a tool-guided synthesis strategy in early drug discovery.^73^

#### 4.1.4 Thioester exchange

**Fig. 10:**
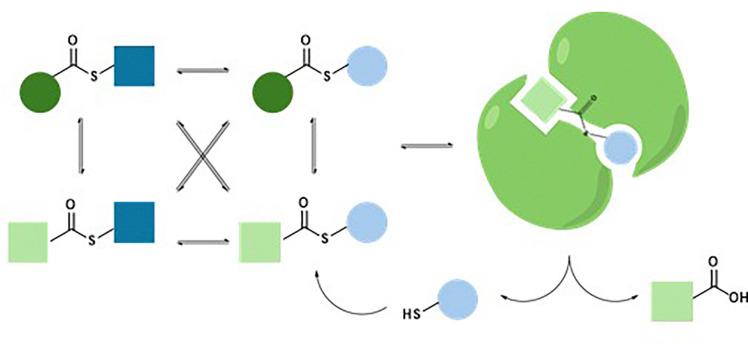
General scheme of a dynamic thioester library of hydrolases. Libraries based on substrate compatibility and catalytic self-screening.

Ramström and colleagues employed a dynamic thioester library where an enzyme selectively catalyzed the conversion of thioester substrates small enough to fit into its binding pocket. This study screened the libraries against various enzymes, including acetylcholinesterase (AChE), butyrylcholinesterase (BChE), β-galactosidase from *Escherichia coli* (β-Gal), esterase from horse liver (HLE), lipase from *Candida cylindracea* (CCL), subtilisin Carlsberg, and trypsin. This selective, enzyme-driven dynamic combinatorial chemistry (DCC) approach enabled the production of libraries based on substrate compatibility (Fig. 10).

The findings demonstrated the effectiveness of transthiolesterification for generating dynamic libraries and highlighted the significant value of the catalytic self-screening strategy for identifying suitable substrates.^37,74^

#### 4.1.5 Boronic acid/boronate ester exchange

Claridge and colleagues employed boronate ester formation in protein-templated dynamic combinatorial chemistry (DCC) to explore enzyme active sites and facilitate the design of selective inhibitors. Using techniques such as ^11^B-NMR spectroscopy, they elucidated the specific interactions between boronic acids, sugars, and target proteins, including α-chymotrypsin. Their findings showed that boronate-based dynamic libraries allow for specific molecular recognition and active-site selectivity, supporting their use in developing enzyme-targeted inhibitors.^75^

Another remarkable advancement was achieved by Wilkinson *et al.*, who employed boronic acid-mediated DCC to identify potent oxygenase inhibitors using a library screened under physiological conditions. This study optimized boronate ester formation between boronic acids and diols, allowing stable interaction with prolyl hydroxylase domain isoform 2 (PHD2). Utilizing dynamic combinatorial mass spectrometry (DCMS), several boronate esters were identified with binding affinity to PHD2, validated through competition assays, and further structural characterization *via* NMR spectroscopy. The resulting inhibitors displayed IC_50_ values in the low micromolar range.^76^

#### 4.1.6 Hemithioacetal exchange

**Fig. 11:**
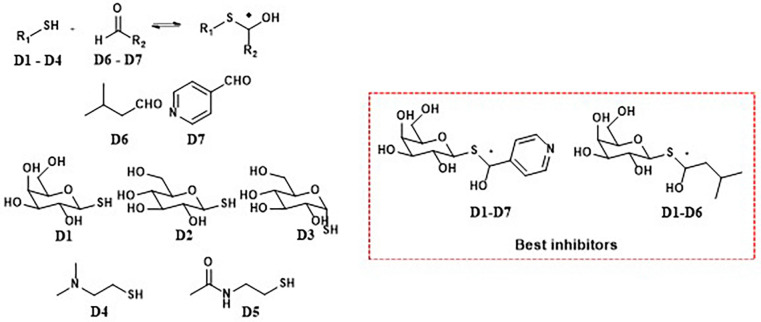
DCL using hemithioacetal exchange for the discovery of β-galactosidase inhibitors.

Hemithioacetals (HTAs) have attracted interest for their practical, reversible binding properties with biological targets under neutral, aqueous conditions. Studies, including work by Caraballo *et al.*, demonstrate that HTAs, formed through the rapid and reversible condensation of thiols with aldehydes or ketones, create a dynamic system that continuously regenerates its components. Using ^1^H STD-NMR, Caraballo *et al.* successfully identified β-galactosidase inhibitors within a dynamic HTA system, highlighting the specific interaction between carbohydrate-based HTAs and the enzyme's binding site (Fig. 11). The fast association and dissociation rates of HTAs allow quick equilibration, making them suitable for biological applications and useful in DCC. These results confirm that specific HTA constituents can act as effective β-galactosidase inhibitors. Inhibition assays, supported by STD-NMR data, revealed that only selected HTAs formed from D1 thiol and aldehydes D6 and D7 combinations significantly slowed substrate hydrolysis, while other system components showed little or no effect.^37,45^ β-Galactosidase inhibitors have therapeutic potential to regulate enzymatic overactivity, such as lysosomal storage disorders and metabolic imbalances.^77,78^

Another study expands on the versatility of dynamic HTA systems by exploring the dynamic asymmetric transformation of hemithioacetal systems. This study highlights the versatility of dynamic HTA systems by exploring lipase-catalyzed γ-lactonization to produce enantiomerically pure 1,3-oxathiolan-5-one derivatives.^79^

#### 4.1.7 Imine exchange

**Fig. 12:**
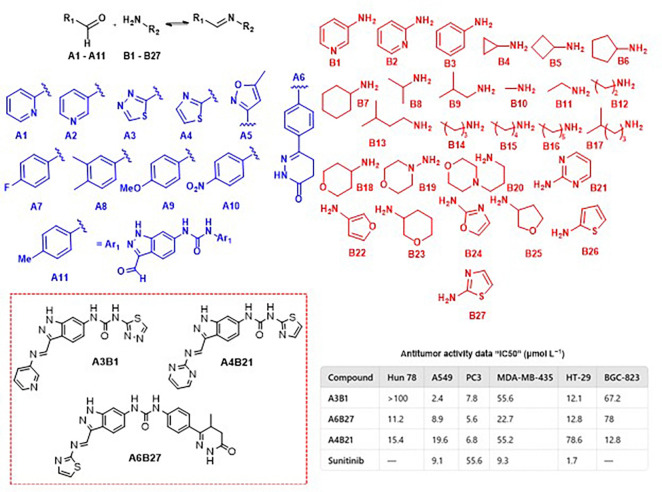
DCL to the discovery of VEGFR-2 inhibitors, IC_50_ values of different human cancer cell lines.

An imine-based DCL targeting receptor tyrosine kinases, specifically VEGFR-2, was developed under thermodynamic control with 102 components. Four compounds (A3B1, A3B2, A6B27, and A4B21) were identified and tested for biological activity. A3B1 and A3B2 showed selective cytotoxicity across several cancer cell lines, with A3B2 being most effective against MDA-MB-435 and HT-29, and A3B1 against A549 and PC3. Additionally, A3B1, A6B27, and A4B21 displayed higher inhibition of angiogenesis in the chick chorioallantoic membrane model compared to the clinical VEGFR-2 inhibitor Sunitinib. These findings confirm their potential as VEGFR-2 inhibitors that block tumor growth by limiting vascular development (Fig. 12).^47^

**Fig. 13:**
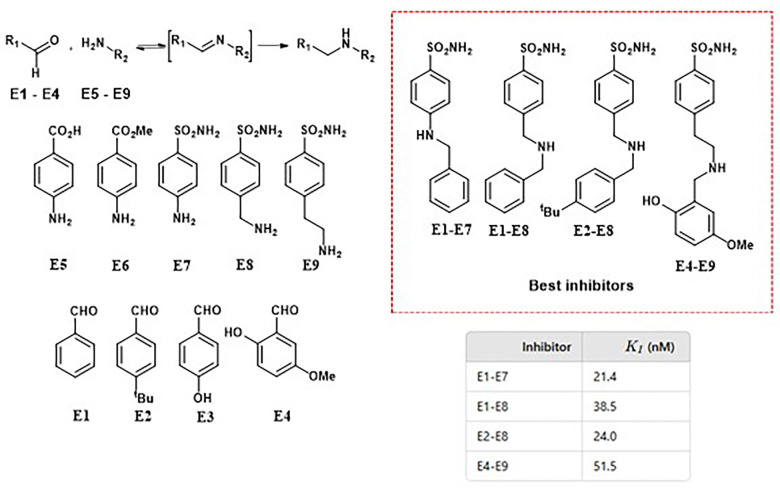
DCL applied to discover inhibitors of human carbonic anhydrase II (hCA II) using sulfonamide building blocks. *K*_i_ values of the best inhibitors are shown.

Dynamic combinatorial chemistry (DCC) has also proven to be a valuable tool for identifying selective inhibitors of human carbonic anhydrase II (hCA II), a crucial metalloenzyme involved in acid–base homeostasis, ion transport, and fluid secretion. A dynamic combinatorial library (DCL) comprising 20 components was generated under thermodynamic control *via* imine exchange. Notably, inhibitors containing sulfonamide groups were selectively amplified, likely due to the strong coordination of the sulfonamide moiety with the Zn^2+^ ion at the enzyme's active site. Screening results revealed a strong correlation between component amplification in the presence of hCA II and their inhibitory potency. The reported inhibition constants (*K*_i_) were in the nanomolar range (Fig. 13).^48^

#### 4.1.8 Oxime exchange

A recent study generated oxime dynamic combinatorial libraries to find potent mGAT1 inhibitors. mGAT1 is the primary GABA transporter^80^ in the central nervous system (CNS) and is crucial for maintaining inhibitory neurotransmission. The authors developed oxime libraries using a racemic nipecotic acid derivative with hydroxylamine functionality, reacting with aromatic aldehydes, and screened in competitive MS binding assays. The combination of competitive MS binding assays with the generation and screening of libraries was first exemplified in pseudo-static hydrazone libraries. Inhibitors with low nanomolar binding affinities and pronounced subtype selectivity favoring mGAT1 were found.^10^

#### 4.1.9 Hydrazone exchange

Meyer's group implemented dynamic combinatorial libraries targeting myeloperoxidase (MPO), combining aromatic aldehydes and hydrazines for MPO binding. MPO, a heme-containing enzyme primarily located in neutrophils, plays a crucial role in immune defense and pathological processes. MPO has become a target for designing anti-inflammatory drugs, and it is being explored in cancer progression.^81^

The inhibitors identified through this process displayed diverse mechanisms, including reversible binding and irreversible inhibition. Some compounds bound strongly to MPO and then became irreversibly inactivated in the presence of hydrogen peroxide. Hydrazones, particularly those derived from hydrazine scaffolds, exhibited high activity due to their unique interactions with MPO's active site. *In vivo* tests using a murine inflammation model demonstrated one dose of irreversible inhibitors can suppress the activity of MPO released after provoking inflammation. This dual functionality—reversible binding under normal conditions and irreversible inhibition in oxidative environments—highlights the potential of hydrazones as therapeutic agents for inflammatory diseases.^82^

#### 4.1.10 *N*-Acylhydrazone exchange

**Fig. 14:**
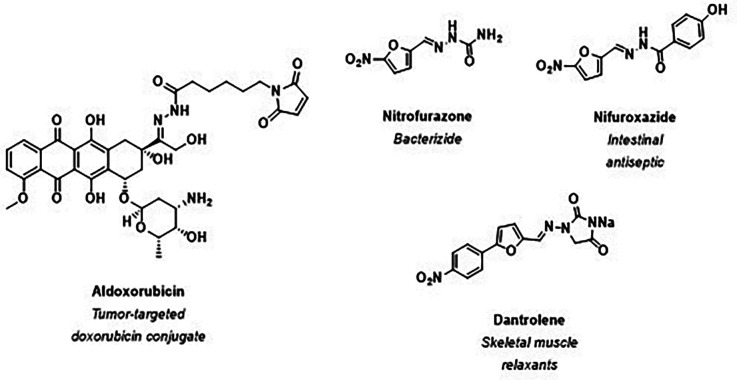
Examples of *N*-acylhydrazone-based FDA-approved drugs.

In recent years, the acylhydrazone exchange has played a central role in protein-directed DCLs. *N*-Acylhydrazone derivatives strongly tend to form stable, biologically active compounds, making them ideal for drug discovery applications. From a total of 117 acylhydrazone patents retrieved, 22 presented pharmacological activities.^83^ The *N*-acylhydrazone scaffold is present in several drugs. For instance, aldoxorubicin (a hydrazone derivative tumor-targeted doxorubicin conjugate) binds covalently to albumin, allowing improved drug delivery and retention in tumor tissues. Besides, nitrofurazone, nifuroxazide, and dantrolene have already been approved for clinical use, demonstrating their therapeutic potential (Fig. 14).^83,84^

Hirsch and coworkers screened a DCL against the 14-3-3 protein to find protein–protein interaction modulators. This protein is crucial in signal transduction, apoptosis, and cell cycle regulation. Two key hits were identified from libraries of acyl hydrazones, exhibiting low micromolar binding affinities for 14-3-3(*ζ*), with dissociation constants (*K*_d_) of 16 μM and 15 μM, respectively. These compounds showed noncompetitive binding behavior since the amplification factors remained consistent with both 14-3-3 alone and its complex with synaptopodin. Synaptopodin is a known ligand of 14-3-3 that binds to its phosphorylation pocket.^11^

In 2019, Perez-Fernández and colleagues reported the first activator of synapse in an animal model of Alzheimer's disease that came from a neuronal calcium sensor 1- directed DCL.^14^ The top compound was amplified from a library of one aldehyde and five acylhydrazides at low temperatures. The compound exhibited affinity to the protein in the micromolar range. The compound's mode of action was studied using Co-immunoprecipitation (Co-IP), NMR, and X-ray crystallography. *In vivo* studies showed the capacity of the compound to promote protein–protein interaction and regenerate synapses.^14^

In an additional case, in a DCL targeting cholesterol esterase (CEase), 18 acylhydrazone derivatives were generated, and two compounds were selectively amplified. The most potent inhibitor showed strong CEase activity with an IC_50_ of 0.36 μM and over 120-fold selectivity against acetylcholinesterase (AChE). The compounds could have potential applications in regulating cholesterol metabolism and addressing related metabolic disorders.^54^

**Fig. 15:**
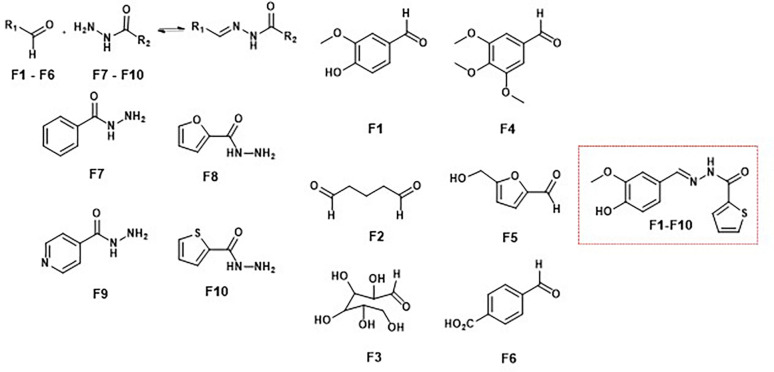
Antiparasitic agents derived from a DCL targeting the bromodomain protein TcBDF3 in *Trypanosoma cruzi*.

Furlan and coworkers reported DCLs targeting the bromodomain protein TcBDF3, a transcriptional regulator in *Trypanosoma cruzi*, highlighting the efficacy of this approach in identifying potent antiparasitic agents. Using acylhydrazone formation under mild conditions with aniline as a catalyst, the DCL adapted its composition in the presence of TcBDF3. This led to the amplification of hydrazone F1–F10, a compound with a *K*_d_ of 1.7 μM for TcBDF3's acetyl-lysine recognition pocket (Fig. 15). F1–F10 also exhibited selective antiparasitic activity, sparing mammalian cells and showing reduced toxicity when TcBDF3 was overexpressed in the parasite.^59^

In 2024, libraries directed at 4-diphosphocytidyl-2*C*-methyl-d-erythritol kinase (IspE) from *Mycobacterium tuberculosis* and the ECF-PanT transporter in *Streptococcus pneumoniae* have revealed inhibitors with potential applications in tuberculosis and bacterial infections, respectively.^16,18^

Ghinet and colleagues reported on the design of libraries targeting the RAGE receptor in inflammatory diseases. They employed microscale thermophoresis (MST) to evaluate the affinities of the DCC hits for the RAGE receptor. The lead compound inhibited RAGE with an IC_50_ of 30 μM, exhibited no cytotoxicity, and maintained cell viability at a concentration of 100 μM, in contrast to the drug Azeliragon.^15^

#### 4.1.11 Disulfide exchange

Mahler and colleagues reported the discovery of *Echinococcus granulosus* thioredoxin glutathione reductase inhibitors by designing a library to target the catalytic site on Trx glutathione reductase. The thioredoxin glutathione reductase (TGR) is a crucial enzyme in the *Echinococcus granulosus* parasite's redox metabolism. The parasite causes globally endemic diseases—cystic echinococcosis and alveolar echinococcosis.^85^

The authors described 5-thio-2-nitrobenzoic acid (TNB) as a non-covalent anchor fragment in a DCL templated by *E. granulosus* TGR. HPLC identified the heterodimer of TNB and bisthiazolidine.^86^

**Fig. 16:**
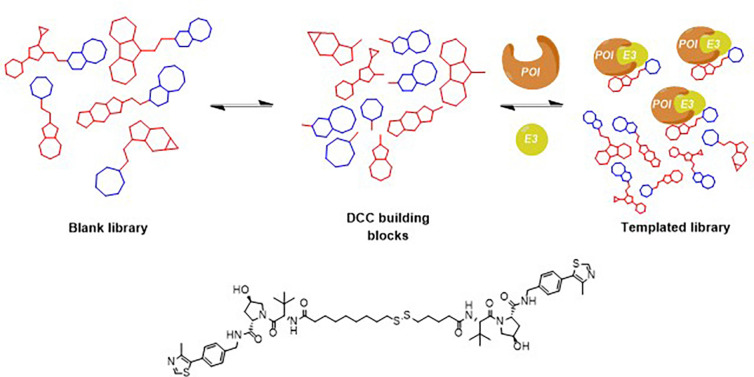
General scheme of a DCL adapted to the PROTAC screening. It is based on ternary complex-directed DCC approach for selecting ternary complex-inducing PROTACs. A potent mediator of ternary complex formation was identified, leading to rapid depletion of pVHL30 levels in cells.

The PROTAC strategy has also been explored using dynamic combinatorial chemistry. In 2024, Ciulli and coworkers demonstrated a proof-of-concept approach that employs thiol-disulfide exchange and the VHL2 ternary complex as a template. Their method enabled the selective amplification of potent homo-PROTAC degraders from dynamic libraries by enriching compounds that promote stable ternary complex formation. This enrichment correlated well with cellular degradation activity and effectively discriminated against non-degrading or monomeric species. While currently applied to VHL, this strategy shows promise for broader use with other E3 ligases and bifunctional proximity-inducing molecules, potentially accelerating hit discovery in early-stage PROTAC development. (Fig. 16).^87^

In another example, a proteomics-based data analysis workflow was integrated with a DCC approach using a combinatorial library of d-peptides in the presence of a Cys-modified peptide ligand targeting programmed cell death 1 (PD-1). This allowed the identification of heterodimeric peptides amplified by the PD-1 protein through disulfide linkage. The amplified peptide dimers were synthesized and confirmed to bind PD-1 and disrupt its interaction with PD-L1 (programmed death-ligand 1), although with no significant improvement in potency compared to the parent peptide. Blocking the PD-1/PD-L1 interaction with immune checkpoint inhibitors is a key strategy in cancer immunotherapy.

Despite challenges such as library size, redox conditions, and PD-1's difficult binding surface, this method demonstrated a powerful and efficient approach for identifying affinity ligands from complex peptide libraries, highlighting the potential of proteomics workflows to streamline high-throughput protein-directed combinatorial screening.^88^

### 4.2 Protein-directed polymer-scaffolded DCLs

This section discusses the use of polymer scaffolds to build DCLs to target proteins of interest. The examples found to meet these criteria have been classified into three groups based on the similarity of the building blocks used in the DCLs. The preferred reversible chemistry utilized was imine and acylhydrazone exchange.

The first group comprises examples of DCLs made of dimethylacrylamide-based polymer scaffolds, solid-supported templates, and lectins, amongst other components. The second group of articles focuses on using amines, aromatic aldehydes, and bi-functionalized polyethylene glycols to prepare the libraries. The last group expounds on using aldehyde-functionalized linear poly(glycidol) and galactose-derived acylhydrazide derivatives to elaborate the dynamic combinatorial libraries, using various enzymes as templates.

#### 4.2.1 Dimethylacrylamide-based polymer scaffolds

**Fig. 17:**
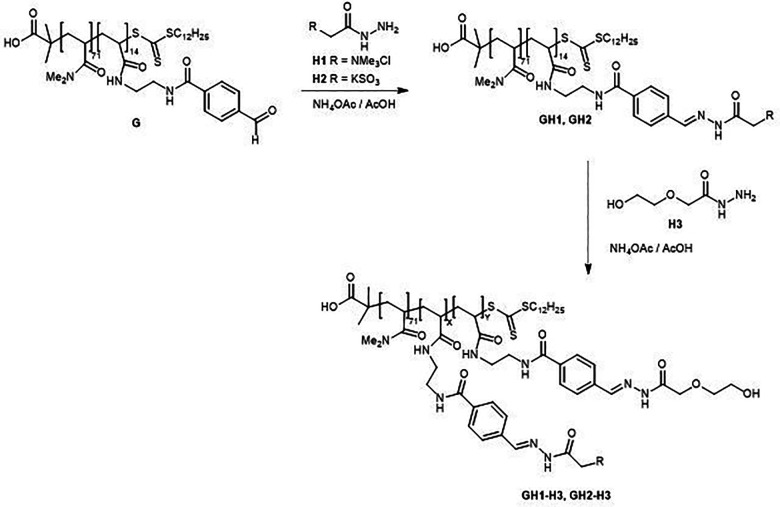
Set up of the polymer-scaffolded dynamic combinatorial library.

Mahon *et al.* describe in these two articles the preparation of polymer-scaffolded dynamic combinatorial libraries (PS-DCLs) in an aqueous solution using reversible conjugation of two different acylhydrazides to aldehyde-functionalized dimethylacrylamide-based polymer scaffolds (Fig. 17).^89,90^

In this study, ^1^H NMR was used to analyze the PS-DCLs, which proved to adapt their composition in response to the addition of three macromolecular template species, specifically poly(sodium-4-styrene sulfonate), bovine serum albumin, and bovine trypsin. It is worth noting that as the molecular weight of the polymer scaffold increased, a linear decrease in the amplification of the preferred residue upon template addition was observed. Likewise, these authors reported a linear relationship between the molar weight percentage of the aldehyde functionalized monomer and the extent of amplification of the preferred residue upon template addition.

The polymer-based dynamic combinatorial library was prepared by a reaction between acylhydrazides and an aldehyde-bearing polymer. Further addition of a macromolecular template induced the re-equilibration of the PS-DCL, preferably incorporating those residues that interacted favorably with the template at the expense of other library members.

Because of the overlap of key signals corresponding to conjugated residues, the residual composition of the PS-DCL could not be determined directly by ^1^H NMR spectroscopy. Instead, this technique was used to detect the relative concentrations of unconjugated hydrazides H1 and H2 in the system, thus enabling the indirect monitoring of the relative composition of the polymer scaffold. The DCLs were allowed to reach equilibrium overnight. They were analyzed by ^1^H NMR during 48 h, showing that H1 and H2 were present in solution at equal concentrations, suggesting they were conjugated to the polymer scaffold in equal proportions. This demonstrated that in the absence of other species, the polymer scaffold showed no preference for condensation with hydrazides H1 and H2. These authors also reported that a greater amplification of the preferred residue upon the polymer scaffold was observed when the surface area : volume ratio of the polymer globule in solution was increased. Also, they could check that at higher template concentrations, templates interacted with unconjugated acylhydrazides, in addition to components of the PS-DCL. The authors proposed that the observed templating effect results from multivalent interactions between functionalized polymers and macromolecular templates.

The same authors have also reported the use of solid-supported templates for the convenient isolation of the best-binding fraction of the library^91^ and a conceptually new approach to the design of macromolecular receptors for lectins.^92^ Commercially available ion exchange resins, such as the quaternary ammonium-functionalized Amberlyst and the sulfonate-functionalized Amberlite, were used as solid-supported analogs of charged macromolecular solution-phase templates. In this case, the composition of PS-DCLs was also monitored indirectly through ^1^H NMR analysis of the methylene protons of the residual unconjugated hydrazides in the solution. The authors hypothesized that library members interacting strongly with the template would eventually get bound to its surface and could, therefore, be isolated from the rest of the library, thus enabling the isolation of macromolecular receptors from solid-supported species of interest. Interestingly, it was also reported that, upon removal of the template, the PS-DCLs re-equilibrated to their original, untemplated composition, thus confirming that changes in the composition of the PS-DCLs are a consequence of a thermodynamically-controlled templating process at supramolecular level.

Regarding DCLs using lectins, the same authors describe the elaboration of carbohydrate-functionalized PS-DCLs in an aqueous solution by the reversible condensation of carbohydrates bearing acylhydrazide functionalities in their aglycone onto an aldehyde-functionalized polymer scaffold. Carbohydrate-binding proteins (lectins) are paramount to cellular recognition and are often implicated in bacterial and viral infection.

In this work, ^1^H NMR analysis of the anomeric protons of galactose and mannose was used to measure the relative concentration of these unconjugated residues in solution, thus allowing the indirect calculation of the relative proportion of each carbohydrate on the polymer scaffold. In this work, using 96-well plates to immobilize lectins is also described as a strategy to generate the so-called ‘temptation-vessels,’ which enabled the isolation of the best binding fraction of the PS-DCL. They pointed out that this strategy could be used in high-throughput techniques to discover macromolecular receptors using PS-DCLs.

#### 4.2.2 Bi-functionalized polyethylene glycol-based polymers

Zhang *et al.* reported an exponential activation of bovine carbonic anhydrase (bCA) in aqueous solutions in the presence of dynameric host matrices based on imine bond formation.^93^ The dynamers were prepared using 1,3,5-benzene-trialdehyde (BTA) (I1), several amines (J2–J7) (including two chiral amines), and the water-soluble linker poly(ethylene glycol)bis(3-aminopropyl) terminated (EG) (I2, *M*_n_ ∼ 1500 g mol^−1^).

**Fig. 18:**
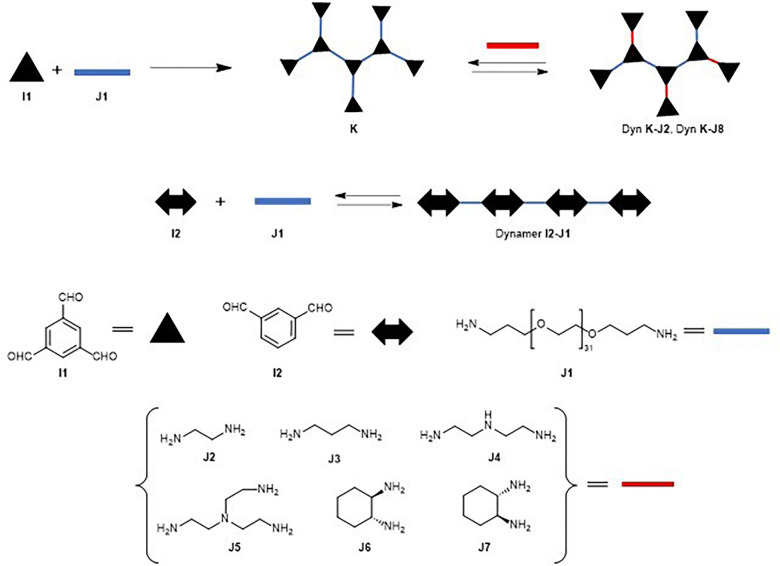
Formation of dynamers from BTA, polyethylene glycol linker and amines.

The dynamer I2–J1 lacking amino components was also prepared from isophthalaldehyde J1 and I2 as a reference to test the effect of the polyethylenglycol linker (Fig. 18). Techniques used included UV/vis for studies of the activation effects of the dynamers, circular dichroism (CD) and fluorescence spectroscopy for binding studies between dynamers K–J6, K–J7 and I2–J1, and bCA. It is worth noting that the CD studies showed selective structural changes in the secondary structure of bCA upon dynamic encapsulation in an aqueous solution. The longer activation time required for one of the dynamers could be explained by the various interaction patterns observed in CD spectra for the dynamers with opposite chirality. The authors concluded that the asymmetric secondary structure of the dynamers somehow induced a chiral microenvironment for the enzyme, which resulted in the structural change of the bCA itself.

The same group reported a straightforward strategy for activating carbonic anhydrase through dynamic encapsulation by adding multivalent amide dynamers into the enzyme reaction solutions.^94^ The authors also reported that they had previously shown that this strategy is functional for DNA binding and its self-adaptive transfection.^95–98^

The libraries were followed by DOSY NMR experiments, fluorescence, and UV-vis spectroscopy.

In addition to its physiological functions, carbonic anhydrase also catalyses other reactions, such as the hydrolysis of *p*-nitrophenylacetate (*p*-NPA), which can be quickly followed by UV-vis spectroscopy.^99,100^ In this work, increasing amounts of polymers were added to the reaction solutions in the UV cuvette containing a fixed concentration of bCA, *p*-NPA in PBS pH 7.0 buffer. The interactions between dynamer and enzyme are mainly controlled through polyethylene glycol matrix-enzyme contacts, whereas the amide/amino functionalized heads contribute mostly to encapsulation/proton shuttling activation effects. The authors conclude that directly adding interactive functional dynamers can enhance enzymatic activity.

In another work by these authors, it is stated that amines with a lower enzyme-directed incorporation ratio to the dynamic constitutional frameworks (DCFs) caused significant enzyme activation effects. Kinetic studies of the enzyme confirmed this activation effect, thus providing information about the enzyme/substrate interactions upon the addition of DCFs, leading to a strategy of self-optimization of the enzyme microenvironment for better catalytic performances.^25^

#### 4.2.3 Functionalized linear poly(glycidol) dynamers

**Fig. 19:**
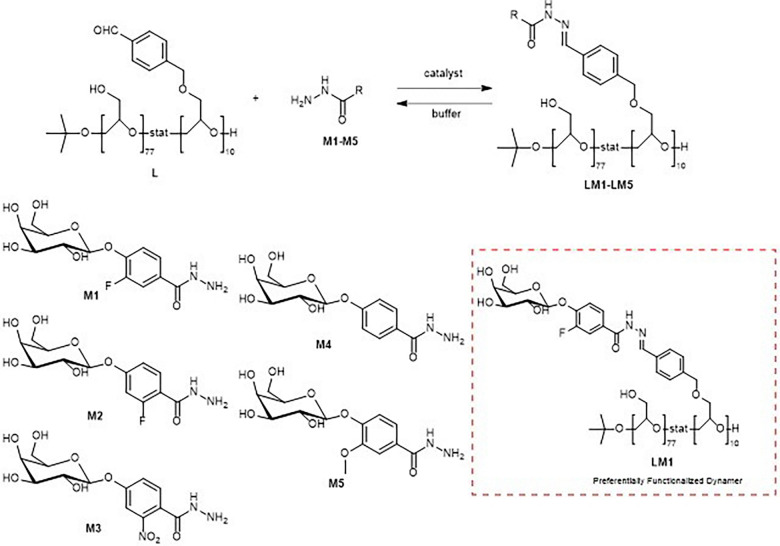
PS-DCL composition using galactose-derived hydrazides as building blocks.

J. Xu *et al.* described the formation of a DCL *via* condensation between aldehyde-functionalized linear poly(glycidol) (L) and galactose-containing acylhydrazide derivatives (M1–M5) (Fig. 19).^23^ Pentameric *E. coli* heat-labile enterotoxin B subunit (LTB) was then applied as an external stimulus, which induced amplification of a particular acylhydrazone side chain that was used in turn to prepare a multivalent LTB inhibitor. The synthesized multivalent inhibitor LM1, containing only the amplified ligand, proved 60 000-fold more potent than its monovalent reference in the binding affinity evaluation. HPLC was used to analyze the DCL. The chromatograms showed no dominant constituent, thus indicating that an average polymer side chain distribution is reached at equilibrium. LTB is known to bind to ganglioside GM1 headgroups bulging out from cells of the gastrointestinal lumen and eventually enables the entrance of toxins into cells.^101^ This binding between LTB and GM1 is one of the highest protein–carbohydrate affinity interactions, making it an interesting target for discovering efficient monovalent inhibitors. This combination of dynamic combinatorial chemistry and polymer-based multivalent inhibitor development protocol has proved a valuable and efficient method for discovering new lead compounds for protein targets.

The same authors described the generation of a polymer-based DCL using aldehyde-functionalized linear poly(glycidol) and hydrazide derivatives as initial building blocks.^102^ Likewise, HPLC was used to indirectly analyze the polymer side chain distribution in equilibrium after adding the template. When combined with tetrameric acetylcholinesterase (AChE), a specific type of acylhydrazone side chain was amplified and then used for the synthesis as a multivalent AChE inhibitor. The multivalent inhibitor proved to have better bioactivity than its monovalent ligand and the commercial reference edrophonium chloride. AChE is a pivotal enzyme that rules signal transmission in nerve cells and is involved in various diseases such as glaucoma and myasthenia gravis. A major form of AChE in mammals is AChE tetramer.^103,104^

The same protocol is applied, but butyrylcholinesterase (BChE) instead of acetylcholinesterase is used to discover a BChE inhibitor with better activity than the commercial BChE inhibitor tacrine. The selectivity between BChE and AChE was also tested.^13^

### 4.3 Nucleic acid-directed DCC (NA-D DCC)

Nucleic acids are highly dynamic biopolymers that play significant roles in various biological processes, such as transcription and translation. In addition to storing genetic information (DNA) and decoding it (RNA), nucleic acids perform architectural, catalytic, and regulatory tasks in the cell. To implement these functions, they must adopt different configurations beyond the conventional duplex or single-stranded RNA shape.^105^ DNA folds into a non-B-form secondary structure under certain conditions, such as hairpin,^106^ triplex,^107^ G-quadruplex,^108^ left-handed Z-DNA,^109^*etc.* In addition, non-canonical RNAs such as microRNA (miRNA), riboswitch, ribozymes, and short interfering RNA (siRNA) can act as endogenous regulators of cellular functions.^110–112^

Therefore, targeting nucleic acids extends the human genome's ‘druggable’ landscape and complements current protein therapeutic approaches in drug discovery.

This part focuses on specific nucleic acid templates, such as double-stranded DNA (dsDNA), G-quadruplexes, and RNA hairpins and loops.

#### 4.3.1 Metal–ligand exchange

Miller and coworkers described a DCL using zinc salicylaldimine complexes to target double-stranded DNA (dsDNA). This system formed a DCL by self-assembling bis(salicylaldiminato)zinc complexes in an aqueous solution. When presented with an immobilized DNA receptor, the equilibrium shifted to amplify the most potent binder, a zinc complex with an apparent dissociation constant of 1.1 μM. Control experiments confirmed that the selection was driven by specific interactions with the DNA receptor rather than nonspecific binding to the affinity matrix. This approach demonstrates the potential of DCC for generating selective ligands for nucleic acids, with the receptor acting as a template to drive the synthesis of its optimal binder.^41^

**Fig. 20:**
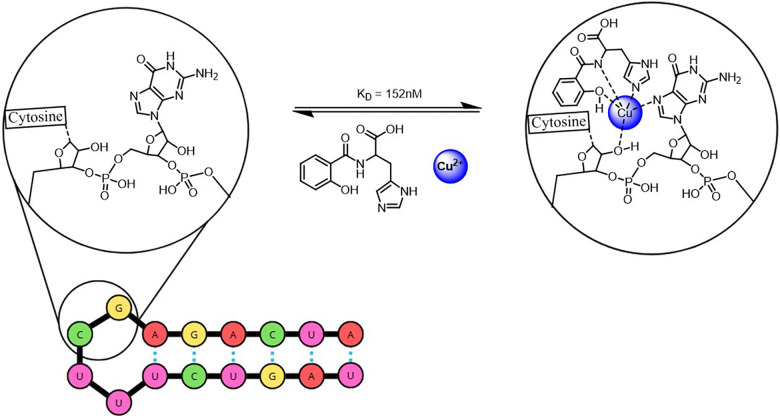
Bis(salicylaldiminato)copper complexes formation for RNA hairpin targeting.

The same group reported a DCL of bis(salicylaldiminato)copper complexes used to target an RNA hairpin derived from the GTP-binding P7 helix of a group I intron. In the presence of the RNA receptor, the equilibrium selected the Cu(ii) complex represented in Fig. 20, which exhibited a dissociation constant of 152 nM and remarkable selectivity, binding the RNA hairpin more than 300-fold more tightly than the homologous DNA sequence. This selectivity likely arises from RNA-specific features, such as the ribosyl 2′-OH group, which can coordinate directly with the metal center. This study highlights the ability of RNA to template ligand selection, offering a promising strategy for identifying selective high-affinity RNA-binding molecules.^113^

#### 4.3.2 Imine exchange

Goodwin and Lynn explored template-directed dynamic covalent chemistry in DNA systems. DNA trimers functionalized with aldehyde and amine groups showed that imine-linked hexamers could be formed selectively in the presence of complementary DNA templates. Upon reduction with sodium cyanoborohydride, stable amine-linked hexamers were obtained. The presence of the template significantly increased hexamer yields, especially at lower temperatures, where template stability was enhanced. Moreover, this system exhibited a remarkable ability to discriminate against non-complementary substrates, achieving up to 30-fold selectivity at 0 °C. This pioneering work laid a critical foundation for applying imine bonds in dynamic combinatorial chemistry (DCC), particularly in nucleic acid systems, by highlighting the interplay between thermodynamic control and template guidance.^114^

Rayner and colleagues explored two complementary approaches to applying imine-based DCC to the discovery of oligonucleotide ligands targeting nucleic acids.

**Fig. 21:**
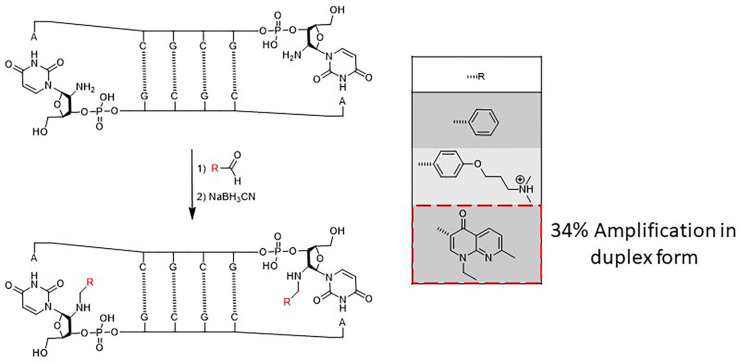
Dynamic combinatorial library using imine-bond DCC on modified oligonucleotides, with *in situ* selection and stabilization of the best-binding conjugates on the duplex form.

Their first study employed imine exchange between 2′-amino-nucleotides and aldehydes to generate a dynamic library of conjugated oligonucleotides (Fig. 21). This strategy enabled the *in situ* formation of diverse imine-linked constructs capable of interacting with RNA and DNA targets. The study demonstrated that selective amplification of the best-binding conjugates occurred upon interaction with the target, highlighting the potential of imine-based DCC in nucleic acid recognition. At the same time, the subsequent conversion of imine bonds into stable secondary amines ensured that the selected ligands retained their binding properties, further validating this methodology as a tool for oligonucleotide ligand discovery.^115^

In a subsequent investigation, Rayner's group refined this approach by constructing a dynamic combinatorial library of mono- and bifunctionalized oligonucleotides to explore sequence-specific recognition. This library incorporated a broader range of building blocks, enabling a more comprehensive assessment of ligand–target interactions. They identified ligands that exhibited enhanced specificity for double stranded RNA motifs by employing a selection process driven by target-induced amplification. Notably, this work extended beyond simple imine exchange to include conjugation strategies that increased ligand stability and selectivity, reinforcing the versatility of DCC for oligonucleotide modification.^116^

Another study by Rayner and colleagues highlighted the adaptability of this approach to triplex-forming oligonucleotides (TFOs). Using a central 2′-aldehyde-modified uridine within an RNA construct, they applied imine exchange with a diverse library of amines. This methodology identified ligands stabilizing DNA triplexes by selectively amplifying conjugates containing polycationic chains, such as tris(2-aminoethyl)amine. These findings highlight the importance of electrostatic interactions in determining ligand selection. They showed that DCC could identify triplex ligands with improved stability and specificity, even at neutral pH—a challenging condition for conventional TFOs.^117^

**Fig. 22:**
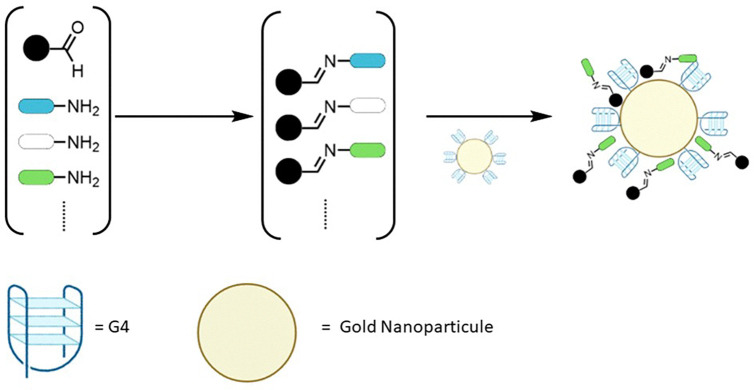
Imine-based DCC, in conjunction with G4, is attached to gold nanoparticles.

G-quadruplex structures are guanine-rich sequences of significant interest due to their regulatory roles in telomere maintenance, transcription, and genome stability. Their Hoogsteen hydrogen bonds and unique π-stacking surfaces offer exceptional recognition potential. Dash and colleagues reported an example where the G-quadruplex DNA (G4-DNA) template was conjugated to gold-coated magnetic nanoparticles.^27^ This setup facilitated the selection of high-affinity ligands from imine-based DCLs composed of carbazole aldehydes and a diverse amine library. The magnetic nano templates simplified ligand separation and allowed for iterative DCC experiments. Among the library members, one ligand exhibited exceptional selectivity and binding affinity for G-quadruplex DNA over duplex DNA, with a Δ*T*_m_ of +23.4 °C in FRET melting assays. This ligand also demonstrated potent anticancer activity by stabilizing G-quadruplex structures in the c-MYC promoter and downregulating its expression. Dash's work showcased the power of imine-based DCC in conjunction with advanced templating strategies to identify biologically relevant ligands (Fig. 22).

**Fig. 23:**
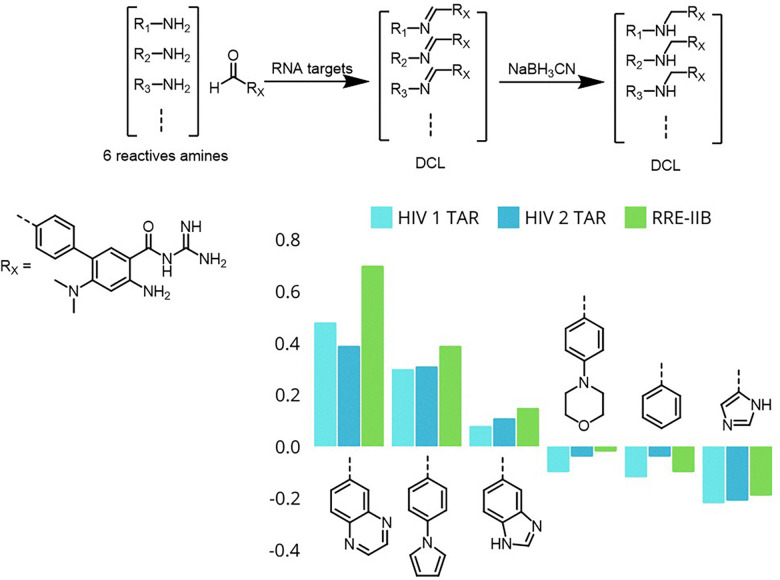
Dynamic combinatorial library of imine-linked ligands based on an aldehyde-modified amiloride scaffold for the targeted selection of HIV-1 TAR, HIV-2 TAR, and RRE-IIB RNA.

Hargrove expanded the scope of imine-based DCC to solution-phase systems targeting RNA, addressing challenges posed by RNA's structural complexity and dynamic nature.^26^ Using an aldehyde-modified amiloride scaffold, they generated a dynamic library of imine-linked ligands screened against HIV-1 TAR, HIV-2 TAR, and RRE-IIB RNA constructs. The amplification of specific ligands correlated well with their binding affinities, which were determined through fluorescence assays and surface plasmon resonance. Importantly, Hargrove demonstrated that this approach could differentiate between closely related RNA targets, highlighting its potential for rapidly discovering selective RNA-binding ligands without requiring high-resolution structural data (Fig. 23).

#### 4.3.3 *N*-Acylhydrazone

**Fig. 24:**
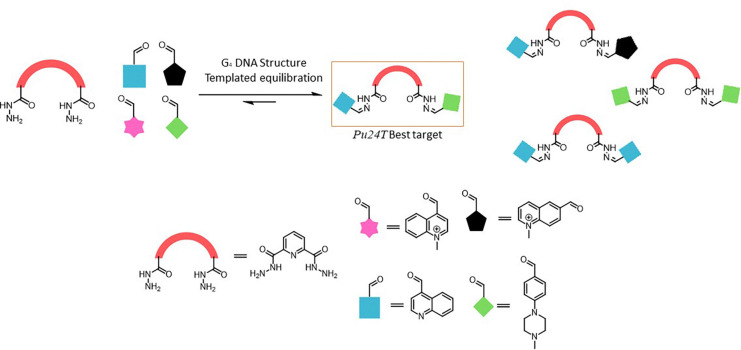
Acylhydrazone-based dynamic combinatorial library for identifying DNA and RNA ligands using a biotinylated pull-down assay.

Granzhan and coworkers employed acylhydrazone-based DCC to identify ligands for G-quadruplex (G4) DNA and RNA, structures that are vital for cellular regulation.^24^ They used cationic aldehydes and dihydrazides to create a library screened *via* a biotinylated pull-down assay, highlighting top ligands that showed a strong G4 stabilization. Subsequent libraries explored structural diversity but faced synthetic challenges, leading to hybrid ligands with moderate G4 affinity (Fig. 24). This work showcases acylhydrazone DCC as a promising selective nucleic acid ligand discovery tool.

#### 4.3.4 Disulfide exchange

**Fig. 25:**
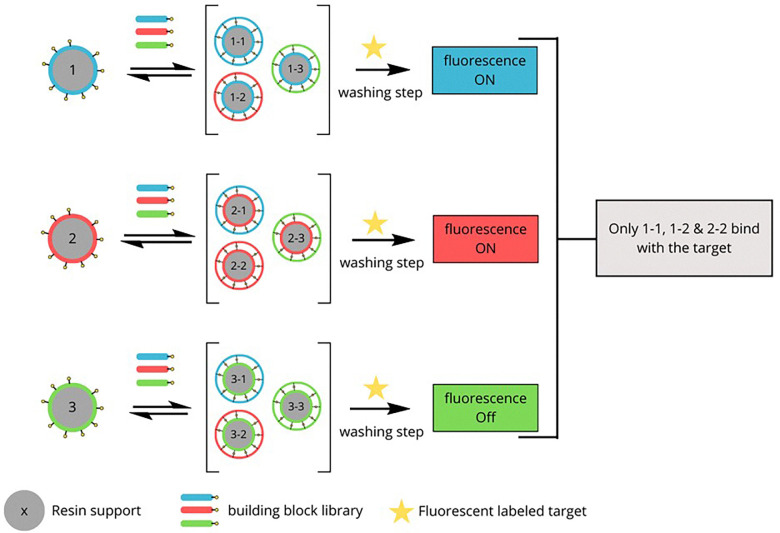
Schematic representation of resin-bound dynamic combinatorial chemistry (RB-DCC) targeting DNA.

Miller's targeting DNA sequences reported the proof-of-concept of resin bound DCC (RB-DCC) (Fig. 25).^29^ Inspired by the octadepsipeptide family of DNA-binding agents, this study used resin-bound building blocks to generate a dynamic library of potential ligands through disulfide exchange. A fluorescently labeled DNA sequence acted as the target, allowing selective enrichment of high-affinity binders. Fluorescent beads are identified under microscopy, and the bound ligands are cleaved photolytically for subsequent analysis by mass spectrometry. The results identified dimeric compounds with notable affinity for the DNA target. Dissociation constants (*K*_d_) were measured to be in the micromolar range, confirming strong and specific binding. This method combines the high-throughput potential of combinatorial chemistry with a straightforward, spatially resolved selection process, eliminating the need for exhaustive solution-phase analysis.

**Fig. 26:**
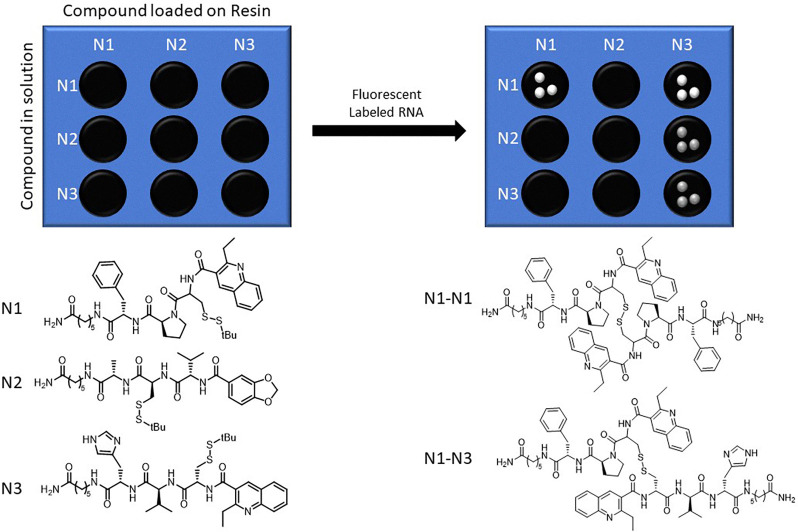
Determination of the highest-affinity ligands for HIV-1 frameshift regulatory stem-loop RNA using RB-DCC. White dots are related to high intensity fluorescence and grey dots are related to mild intensity fluorescence.

Following this example, Miller's team used RB-DCC to target disease-relevant RNA sequences.^118^ Their first RNA-focused application identified ligands for the HIV-1 frameshift regulatory RNA. Using a library of 11 325 theoretical members, this study demonstrated that resin-bound and solution-phase components compete dynamically, ensuring the selection of the highest-affinity binders (Fig. 26). RB-DCC experiments revealed that dimers N1-N1 and N1–N3 were enriched in the presence of the RNA stem-loop. The absence of signal for N3-N1 highlighted the influence of orientation in binding. This result illustrates a key feature of RBDCC: competition between solution-phase and resin-bound species, where only ligands formed and retained on resin generate a detectable signal. The data suggest N1-N1 forms a higher-affinity complex on-resin, enabling its selective identification. Here, selected ligands exhibited strong sequence selectivity, with top candidate N1-N1 showing dissociation constants (*K*_d_) in the micromolar range for the target RNA and negligible affinity for unrelated sequences.

Another application of RB-DCC was described targeting RNA-mediated myotonic dystrophy type 1 (DM1).^119^ In this example, RB-DCC was employed to screen the same extensive library against (CUG) repeat RNA, a hallmark of DM1 pathology. Fluorescently labeled (CUG)*n* RNA was used to identify resin-bound ligands, followed by cleavage and mass spectrometry analysis. The process yielded four key components, which were recombined into 16 possible homo- and heterodimers for further evaluation. The screening revealed that only two dimers exhibited an affinity for (CUG)*n* sequences, with dissociation constants of approximately 2 μM determined by fluorescence titration. These ligands also disrupted the toxic RNA–protein interaction with MBNL1, a key factor in DM1 pathology.

Balasubramanian and colleagues extended the scope of disulfide-based DCC by targeting DNA G-quadruplexes, structures of significant interest due to their roles in telomere maintenance and transcriptional regulation. In one of their first examples, they designed a library combining hydrophobic acridone units and peptide fragments with quadruplex-binding potential.^120^ The library, equilibrated with a human telomeric G-quadruplex, selectively amplified acridone–peptide heterodimers and peptide homodimers. These ligands exhibited enhanced binding affinity for the quadruplex target, as confirmed by surface plasmon resonance (SPR) and fluorescence titration, with dissociation constants in the micromolar range.

A related study explored the specificity of distamycin analogs for DNA quadruplex *versus* duplex structures.^121^ The researchers incorporated pyrrole and imidazole polyamides into a disulfide library to identify ligands with distinct binding preferences. Quadruplex DNA elicited moderate amplification of specific polyamide disulfides, while duplex DNA induced more substantial amplification, reflecting the differential recognition potential of these ligands. Thermal melting experiments further corroborated the selective stabilization of quadruplex or duplex structures by specific library members, emphasizing the utility of DCC in uncovering nuanced binding interactions.

This work also showed the role of competing thiols, such as glutathione, in modulating the equilibrium and enhancing the sensitivity of DCC systems. More precise amplification signals were shown by suppressing self-association among library members, enabling the identification of ligands with high specificity for their nucleic acid targets.

**Fig. 27:**
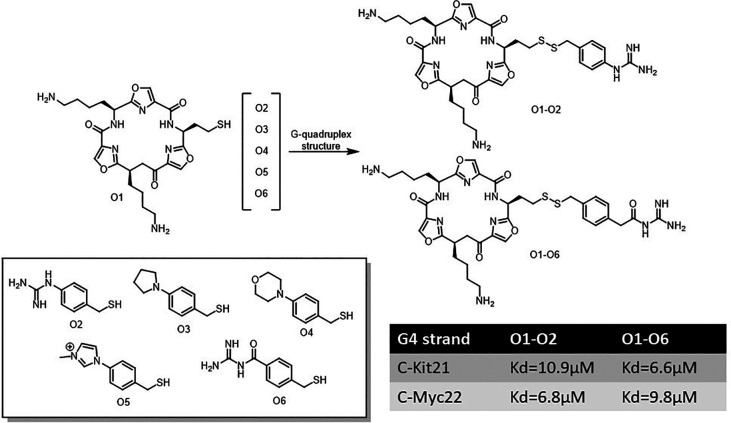
Dynamic combinatorial library based on disulfide chemistry targeting G-quadruplex structures c-Kit21 and c-Myc22, with corresponding dissociation constants for the top binders.

In a later work, Balasubramanian and Sanders employed DCC to investigate recognizing distinct G-quadruplex sequences.^122^ Their system utilized an oxazole-based peptide macrocycle as the scaffold, with libraries of thiol-modified side chains, including positively charged and neutral carbohydrate derivatives. In the presence of quadruplex targets derived from the promoters of oncogenes (c-MYC22 and c-KIT21), the library revealed selective amplification of macrocycle-side chain conjugates. Notably, the patterns of amplification varied between the c-MYC22 and c-KIT21 quadruplexes, highlighting the ability of subtle chemical and stereochemical variations to tune ligand specificity (Fig. 27).

Surface plasmon resonance confirmed that the most amplified ligands bound their respective quadruplexes with dissociation constants in the low micromolar range. The study also introduced carbohydrate-based side chains as promising motifs for quadruplex recognition, showcasing the potential of glycosidic interactions to enhance binding affinity and selectivity.

**Fig. 28:**
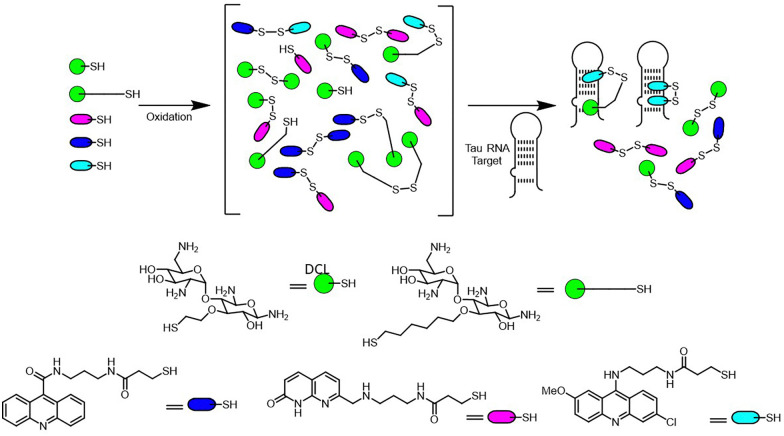
Dynamic combinatorial library targeting the Tau Exon 10 stem-loop RNA, highlighting the selective amplification of acridine–neamine heterodimers and their stabilizing effects on RNA secondary structures.

In 2011, Marchán and colleagues applied DCC to RNA targets such as the Tau exon 10 stem-loop structure involved in tauopathies.^123^ Their library incorporated aminoglycosides and heteroaromatic moieties, which formed disulfide-linked dimers upon equilibration (Fig. 28). The RNA template selectively amplified ligands containing azaquinolone or acridine derivatives, with notable amplification of the acridine–neamine heterodimer. Fluorescence titration and thermal melting experiments confirmed that the most amplified ligands bound with high affinity and stabilized the RNA secondary structure, including disease-associated mutated variants.

Interestingly, the ligands' ability to stabilize the RNA correlated directly with their level of amplification in the DCC experiments. Circular dichroism spectroscopy further demonstrated that the ligands maintained the RNA's overall conformation, ensuring compatibility with the splicing machinery. This highlights template-directed DCC's potential for identifying disease-modifying ligands with therapeutic relevance.

## 5 Conclusions and perspectives

Drug discovery continuously evolves, driven by the demand for more efficient, sustainable, and high-throughput strategies. Protein-directed dynamic combinatorial chemistry (P-D DCC) and nucleic acid-directed dynamic combinatorial chemistry (NA-D DCC) have emerged as powerful approaches for ligand identification. These methodologies streamline drug design by combining *in situ* synthesis with direct target screening.

Target-directed DCC has demonstrated success across various protein and nucleic acid targets, utilizing diverse reversible covalent chemistries such as imine, acylhydrazone, and disulfide exchange. Site-directed strategies have further expanded their applicability by enabling the exploration of previously inaccessible binding pockets. However, challenges remain, including the complexity of large combinatorial libraries, limitations in current analytical techniques, and the long equilibration times that can compromise target stability and hinder accurate screening. Addressing these challenges by integrating biocompatible catalysts into DCC frameworks holds potential for expanding its biological applications.

The future of target-directed DCC lies in advancing biocompatible reactions, enhancing screening technologies, improving real-time analytical methods, and leveraging machine learning-driven library design. These innovations will improve the precision, efficiency, and sustainability of drug discovery, reinforcing DCC as a valuable tool for identifying antiviral, antibacterial, and anticancer agents. Target-directed DCC will continue to support rational drug discovery and biomolecular innovation by overcoming current limitations and refining library design.

## Conflicts of interest

There are no conflicts to declare.

## Data Availability

This review relies on existing published studies, all cited within the manuscript. The data supporting this work can be found in the referenced sources.
